# The Role of New Technologies to Prevent Suicide in Adolescence: A Systematic Review of the Literature

**DOI:** 10.3390/medicina57020109

**Published:** 2021-01-26

**Authors:** Alberto Forte, Giuseppe Sarli, Lorenzo Polidori, David Lester, Maurizio Pompili

**Affiliations:** 1 Psychiatry Residency Training Program, Faculty of Medicine and Psychology, Sapienza University of Roma, 00185 Roma, Italy; giuseppesarli194@gmail.com (G.S.); lorenzo.polidori11@gmail.com (L.P.); 2 Department of Psychiatry and Substance Abuse, ASL Roma5, 00015 Rome, Italy; 3 Psychology Program, Stockton University, Galloway, NJ 08205, USA; david.lester@stockton.edu; 4 Department of Neurosciences, Mental Health and Sensory Organs, Suicide Prevention Center, Sant’Andrea Hospital, Sapienza University, 00185 Rome, Italy; maurizio.pompili@uniroma1.it

**Keywords:** suicide prevention, adolescents, technologies

## Abstract

*Background and objectives*: Suicide in adolescents represents a major public health concern. To date, a growing number of suicide preventive strategies based on the use of new technologies are emerging. We aimed to provide an overview of the present literature on the use of new technologies in adolescent suicide prevention. *Materials and methods*: An electronic search was run using the following keywords: Technology OR Technologies OR APP OR Application OR mobile application) AND (Adolescent OR youth OR puberty) AND (Suicid* OR Self-harm OR self-destruction). Inclusion criteria were: English language, published in a peer-reviewed journal, suicide prevention with the use of new technologies among adolescents. *Results*: Our search strategy yielded a total of 12 studies on the use of telemedicine, 7 on mobile applications, and 3 on language detection. We also found heterogeneity regarding the study design: 3 are randomized controlled trials (RCT), 13 are open-label single group trials, 2 are randomized studies, and 1 is a cross-sectional study. Telemedicine was the most adopted tool, especially web-based approaches. Mobile applications mostly focused on screening of depressive symptoms and suicidal ideation, and for clinical monitoring through the use of text messages. Although telepsychiatry and mobile applications can provide a fast and safe tool, supporting and preceding a face-to-face clinical assessment, only a few studies demonstrated efficacy in preventing suicide among adolescents through the use of these interventions. Some studies suggested algorithms able to recognize people at risk of suicide from the exploration of the language on social media posts. *Conclusions*: New technologies were found to be well accepted and tolerated supports for suicide prevention in adolescents. However, to date, few data support the use of such interventions in clinical practice and preventive strategies. Further studies are needed to test their efficacy in suicide prevention among adolescents and young adults.

## 1. Introduction

Mental health problems and, more specifically, suicide are relevant public health concerns in adolescence [1,2]. Up to 20% of adolescents suffer from a psychiatric disorder [3], and psychiatric disorders account for about 45% of the years lived in disability for those between 10 to 24 years of age [4]. Suicide is one of the major causes of death worldwide among youth, ranking second for the 15–19 age group [5]. Thus, suicide prevention in adolescents should be considered a priority among public health policies [6]. Suicide ideation is strongly predictive of suicide attempts and suicide mortality [7]. Thus, efforts aimed at preventing, detecting, and, eventually treating suicidal ideation might also prevent suicide attempts and deaths from suicide [8].

Nowadays the use of technology is increasing in medical fields and specialties and, even in psychiatry, the concept of “telepsychiatry” is acquiring a more defined identity [9]. Moreover, given the recent public health crisis due to the COVID-19 pandemic, clinicians require new tools for delivering services and preventive interventions [10,11]. Technology is providing new frontiers in psychiatry, and the usual face-to-face suicide risk assessment of patients has now often been replaced with consultations based on digital tools [12,13,14,15,16]. Thus, regarding suicide prevention, fast and effective response to the patient’s needs, avoiding delay, might be implemented using new technologies [12,17].

The focus of the present paper was to investigate some of the existing approaches to suicide prevention based on new technologies and data collection targeted at the adolescent population. Adolescents are avid users of technology. Almost a quarter of adolescents are online constantly and more than 90% are online daily [18]. Adolescents and young adults are technologically savvy, and a large proportion of them own a smartphone or other devices that allow for different types of interactions; phone calls, texts, Facebook messages, Tweets, and blog posts are just a few examples of possible communication channels used to keep social connections and to share ideas, thoughts and emotions. Thus, there is no doubt about the importance of interventions based on new technologies in suicide prevention among youths. A previous systematic review and meta-analysis investigated the effectiveness of digital interventions (focusing on smartphone apps) for the self-management of suicidal ideation or self-harm among both adults and adolescents [19]. However, to our knowledge, this is the first qualitative overview of the current literature regarding the use of a broader range of new technologies specifically developed for preventing suicidal behavior among adolescents. Thus, the aims of the present paper were: (1) to provide a systematic review of available and published studies focusing on interventions based on the use of new technologies and (2) to provide a detailed picture of the currently available preventive strategies using digital tools for adolescents.

## 2. Materials and Methods

### 2.1. Search Strategy and Information Sources

A computerized literature search of the MEDLINE/PubMed, PsycINFO, and EMBASE databases, following PRISMA checklist [20], using the following keywords: (Technology OR Technologies OR APP OR Application OR mobile application) AND (Adolescent OR youth OR puberty) AND (Suicid* OR Self-harm OR self-destruction). Examining references cited in identified reports extended these electronic searches.

### 2.2. Study Selection

**Figure 1:**
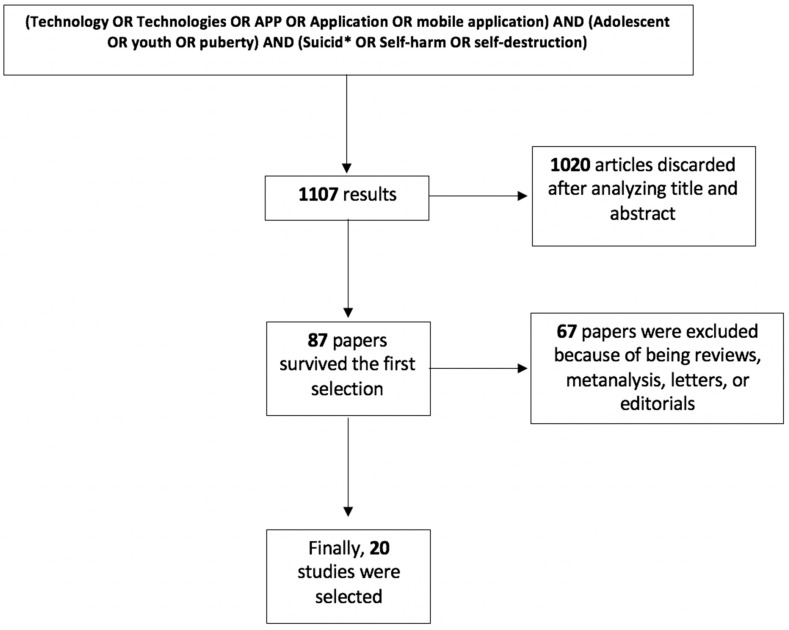
Search strategy.

As shown in Figure 1, a total of 1107 records resulted from the initial database search. The first selection of records was made by two reviewers (G.S. and L.P.) by analyzing the title and abstract of the records, and only research studies focusing on the use of technologies in suicide prevention among adolescents were considered. Overall, 87 studies were first screened and considered potentially relevant for our purpose.

We included original articles published in English in peer-reviewed journals, that focused on suicidal behavior and its prevention with the use of new technologies among adolescents.

We excluded articles not written in the English language, studies of behaviors other than suicidal behavior, papers considering an age-range population different from adolescence (age range 10–25 years) [21], and case reports. At the end of the screening process, 20 articles were included in our qualitative synthesis (Figure 1). Three reviewers selected the studies (A.F., G.S., L.P.). Disagreements in the selection process and/or extraction of data was solved by consensus and involving an additional senior reviewer (M.P.).

### 2.3. Data Extraction

Three investigators (A.F., G.S., L.P.) independently extracted data from the chosen reports. Measures extracted from each study sample included the following: country of origin, years in which data were collected, total sample size, duration of follow-up, the proportion of women, mean age, suicidal behavior (suicidal ideation and/or suicide attempt), psychiatric diagnoses, self-harming behaviors, and concomitant medication.

### 2.4. Risk of Bias within Studies

To assess risk of bias, a quality assessment was completed using the National Institutes of Health Quality Assessment Tool of Controlled Intervention Studies (Table 1), designed to examine study quality according to Cochrane collaboration criteria [22,23].

## 3. Results

### 3.1. Studies’ Selection, Characteristics and Limitations

The 20 papers included in the qualitative synthesis are characterized by great heterogeneity regarding both the study design and the technologies adopted for preventing suicide in adolescence (see Table 2).

Focusing on the study design, 2 studies were randomized controlled trials (RCT) [24,25], 12 were open-label single group trials [26,27,28,29,30,31,32,33,34,35,36,37] 2 were randomized studies [38,39], 4 were retrospective cohort studies [40,41,42,43]. The overall sample consisted of 221,419 adolescents. The concomitant psychiatric diagnosis was included in seven studies; six studies took into account depressive disorders [27,28,30,31,33,38], two autism spectrum disorders [27,42] and two anxiety disorders [31,38]. Only one study [24] included concomitant medication.

Suicidal behavior was investigated differently in the studies; nine studies focused only on suicidal ideation [25,26,28,29,30,34,39,41,43], six on suicide attempts [24,27,32,33,40,42]; and two analyzed both suicide ideation and suicide attempts [36,38]. Given the results obtained from the literature search, three main different technological tools were found: telepsychiatry, mobile health intervention, language detection.

### 3.2. Telepsychiatry

Our search strategy found 11 studies on the use of telepsychiatry as a tool for suicide prevention. In 2016 a British study tested whether a text-messaging intervention to support adolescents who self-harm (TeenTEXT) could be administered by clinicians at child and adolescent mental health services (CAMHS) within the context of everyday clinical practice. Despite the interest by clinicians in using the intervention, they found very limited engagement in practice, and only six patient–clinician dyads were recruited [37]. Thus, the study failed to demonstrate any effect of the intervention in preventing suicide. In the same year, Robinson and colleagues started a pilot study to test the efficacy of a newly designed eight-module Internet-based program, trying to identify suicidal ideation among adolescents (21 secondary school students). At the end of the study, there was a significant reduction in all dimensions that were targeted as outcomes of interest (depression, hopelessness, and suicidal ideation), suggesting that Internet-based programs could have a role in preventing suicide among the youth [28]. Chen and colleagues developed an automated text message intervention using a platform for both depression (EpxDepression) and autism spectrum disorder (ASD; EpxAutism) [27]. They focused their research on depression and autism (with six and three participants, respectively) with two subtypes of the platform: EpxDepression and EpxAutism. The platform utilized an automated system to triage patients into three risk categories based on their responses and alerted clinicians directly when patients met specific risk criteria. EpxDepression detected thoughts of self-harm in patients before their case managers or caregivers were aware of such ideation [27].

In 2018, an Australian study from two mental health services (headspace Camperdown and headspace Campbelltown) tested a new technology for online assessment called the “Mental Health eClinic (MHeC)” [36]. Comparing online assessment and standard assessment in face-to-face services, they found good agreement between the two techniques (68%, kappa = 0.39). The authors found that the online assessment placed a greater focus on the history of mental health problems (*p* = 0.001), as well as any previous suicide planning (*p* = 0.002) and current comorbidity with cannabis misuse (*p* = 0.03) as indicators of the progression of the disease. They concluded that the online assessment process could be a more efficient way of detecting the lifetime severity of the disorders.

A recent randomized trial (n = 110) compared the effectiveness and acceptability of the Case-finding and Help Assessment Tool (YouthCHAT, a self-report, electronic screener of several domains such as drug use, depression, gambling, etc.) with a face-to-face assessment [39]. The results demonstrated that YouthCHAT was a time-saving method, with a mean difference of 8 min 25 s. compared to standard assessment. Moreover, YouthCHAT was found to be an effective and acceptable screener for use in a secondary school youth populations, with similar or significantly higher detection rates than the face-to-face assessment [39].

Dickter and colleagues [30] tested the impact of an Internet-based depression prevention intervention (CATCH-IT) on risk factors for suicide (such as suicidal ideation, hopelessness, low self-esteem, and social isolation). The program consisted of self-guided, online modules based on cognitive-behavioral therapy (CBT) and interpersonal psychotherapy aimed at increasing skills for developing resilience and decreasing vulnerability to depressive symptoms. The authors found a significant change in suicidal ideation in adolescents at risk of depression after using the platform, even with very small effect sizes. Mean suicidal ideation across all participants decreased by 3.3% (*p* < 0.05; d = 0.22) [30]. Interestingly, when they analyzed only those who completed all 14 modules (n = 24), mean suicidal ideation decreased by 8.8%, with a moderate effect size (*p* = 0.01; d = 0.60). Recently, Han et al. investigated whether web conferencing technology-based online focus groups (W-OFGs) are an efficient method to involve young people, who have suicidal thoughts, in suicide prevention programs. They found a high rate of participation (70%) and good acceptability by users [32].

An Australian study analyzed the association between sexting and suicidal behaviors, as well as with several other mental health negative outcomes (body image issues, and information and communication technology [ICT] safety risks, including cyberbullying and late-night Internet use). Milton, et al. [35] run a survey using computer-assisted telephone interviewing (CATI) and found that sexting (both receiving and sending) were significantly associated with reporting suicidal thoughts and behaviors in the past 12 months. Using an ecological study, Runkle and colleagues [29] analyzed the role of crisis text lines during a weather-related disaster in North and South Carolina, USA (Hurricane Florence). The aim was to study the psychological impact of a disaster on youth from an analysis of the variation of crisis text volume before and after the hurricane. The adolescents were seeking help for several problems, including stress and anxiety, depression, and suicidal thoughts. As a low-cost and immediate service, the crisis-texting platform succeeded in providing 24/7 mental health support for the youths. Based on this experience, the authors highlighted how text-based crisis support services could identify the mental health consequences of a disaster along with the measuring the situational awareness in an impacted community.

### 3.3. Mobile Health Interventions

Smartphone applications can be considered to be an evolution of telepsychiatry. Five studies focused on the use of a mobile app to prevent suicide [24,31,33,34,38].

Kennard and colleagues evaluated suicidal behavior using the app BRITE in the ASAP (As Safe as Possible) study [38]. In this study, 66 inpatients were recruited and then monitored using BRITE (suitable for both IOS and Android software). The participants received daily text messages to rate their level of emotional distress (on a scale of 1–5, with 5 being “most upsetting”). Based on their level of distress, participants were offered a range of distress tolerance and emotion regulation skills. However, despite its acceptability, this randomized controlled trial did not detect any substantial clinical effects on either suicidal ideation or attempts; but a non-significant reduction in the rate of suicide attempts among the participants assigned to ASAP plus treatment as usual was observed (hazard ratio = 0.23, 95% CI = 0.05, 1.09).

Grist and colleagues explored the safety, use, and acceptability of the BlueIce app [31]. This mobile phone app was tested for reducing and preventing self-harm among adolescents. Forty participants were recruited from a child and adolescent mental health services (CAMHS), all aged between 12 and 17 and with a history of self-harm. The results from this study showed that BlueIce was helpful and safe in supporting adolescents, and it also showed some effectiveness in managing thoughts of self-harming [31].

There is now a growing number of studies on new mobile interventions that have showed feasibility, even if they have not yet been tested for their efficacy [44].

### 3.4. Language Detection

Four studies adopted language detection as a suicide prevention strategy. In this approach textual features are extracted from online posts (forums, tweets, and other social media) for detecting suicidality. Statistical classification algorithms, using logistic regression, random forest, and support vector machine (SVM) algorithms are applied to disclose patterns and relationships between text features and suicidality [40,41,42,43].

Aladag and colleagues screened 508,398 Reddit posts longer than 100 characters, posted between 2008 and 2016 on SuicideWatch, Depression, Anxiety, and ShowerThoughts subreddits. They chose 785 posts to analyze; textual parts were extracted from posts for discriminating suicidality, then statistical classification algorithms (logistic regression, random forest, and then support vector machine) were applied to detect relationships between the extracted text and suicidality. Four different experiments were conducted trying to discriminate different levels of suicidality among posts. The findings from this study showed that text-mining methods could be used to detect posts with suicidal ideation online, on a real-time base using a Javascript or mobile app library [40]. Grant and colleagues also adopted a text mining approach to analyze 63,232 posts from the social media platform Reddit, specifically the subreddit called r/SuicideWatch [43]. They extracted informal latent topics from online social media expressing suicidal ideations. They first evaluated the latent topics and then compared them to risk factors proposed by domain experts. Thus, they identified specific informal terminology used online and compared it to specific risk factors, to build up new models to detect suicidality.

Brown and colleagues applied a quality text analysis software (ATLAS.ti 7) (N = 52 participants) to detect and investigate expressions related to active suicidal thoughts on Instagram. The language used was then compared to the language emerging from interviews. No differences in activity and language use were found to be associated with acute suicidality, and authors concluded that other machine learning approaches might be more appropriate for detecting suicidality on social networks [41].

Downs and colleagues screened 230,465 documents obtained from a large autism spectrum disorder (ASD) electronic health records system using a natural language processing (NLP) system. They then tested NLP on 500 ASD patients as a tool to evaluate positive and negated suicide intentions. The study showed that NLP could be useful to detect suicidality within the health records of young people with ASD [42].

## 4. Discussion

The use of tele-communication and the Internet has progressed from the use of the telephone in the 1970s and, later, the Internet for crisis intervention [45]. The present literature overview examined all available studies focusing on suicide prevention interventions among adolescents and young adults based on the use of new technologies. Across the 20 included studies, we found that three main technological tools have been tested as suicide preventive interventions: telemedicine, mobile health interventions, and language detection. The results of this overview showed that most of the studies focused their investigation on the adoption of telemedicine in psychiatry for preventing suicide in adolescents. As defined by the American Psychiatric Association, telepsychiatry is defined as “a subset of telemedicine, [which] can involve providing a range of services including psychiatric evaluations, therapy (individual therapy, group therapy, family therapy), patient education and medication management” [9].

Most of the studies we identified were Internet-based interventions, most of which demonstrated good acceptability and satisfaction among users [30,39]. Interestingly, older reports showed less engagement from users [37], suggesting that new generations might be more easily involved in the use of digital tools. However, despite the acceptability of these new digital tools for telepsychiatry, only a few studies demonstrated efficacy in preventing suicide among adolescents through the use of online and telephone-based interventions [27,30]. Of note, telepsychiatry might be considered especially suitable for reaching populations characterized by low engagement with traditional health care facilities, such as adolescents [46]. Moreover, telemedicine is already supporting new promising methodologies in detecting suicidal behaviors among selected populations, such as ecological momentary assessment (EMA) [47,48]. Web platforms might also be involved in school programs aimed at preventing suicide in students [44].

We also found several studies testing different mobile apps for smartphones, especially for screening for depressive symptoms and suicidal ideation [31] and for clinical monitoring through the use of text messages [38]. Results from this review showed that the use of a mobile app is a fast and easy way to reach adolescents, to keep in contact with them and to monitor their suicidal behaviors [38]. Moreover, given the very high rates of suicide attempts and deaths from suicide after discharge from psychiatric facilities [48,49], it seems important to develop new digital tools (such as the app BRITE) for screening and supporting such a high-risk population [38]. A large proportion of suicidal behaviors occur within the first three weeks of outpatient treatment following hospital discharge [50,51], meaning that even rapid referral to outpatient care may only partially alleviate the high rate of suicidal behavior after hospital discharge. Thus, a tailored app can provide a fast and safe tool, supporting and preceding a face-to-face clinical assessment after discharge [38].

A few studies focused on the use of language detection to identify suicidality among social media users. With the rise in sophistication and use of online social networks, suicidal thoughts have been increasingly expressed in online forums, tweets, and other social media, resulting in a vast collection of thoughts and motivations associated with suicide [43]. The challenge consists in developing language detection programs that can reveal suicidal intent from posts on social media. Some of the studies we found focused on Reddit, which is a suitable social media platform because of a specific suicidal subsection and because it allows longer posts [40,43]. Interestingly, language detection can also be applied to electronic health records [42], suggesting future possibilities for studies using large electronic databases. Despite the limited amount of evidence, some authors have already developed algorithms able to recognize people at risk of suicide from the exploration of the language on social media posts, precise and timely enough to promise some clinical effectiveness [52]. However, little thought has focused on useful ways of responding to such online communications when they occur.

Many of the studies in this review suffer from significant theoretical and practical limitations. Most studies on commercial apps have not adhered to rigorous evidenced-based procedures, and the results usually lack validation by medical standards. Moreover, no systematic assessment has been conducted on the relative validity and reliability of active self-reporting and passive data collection, which is one of the major advantages of using digital tools such as smartphone or web platforms. Notably, most of the studies focused on patients individually, whereas their social context (such as family members and caregivers) can be equally important in preventing suicide among adolescents. Moreover, several ethical issues arise (such as privacy and social media suicide prevention protocols) from the use of new technologies, especially language detection, that need to be addressed before their use for screening and monitoring. Furthermore, the cultural implications of the implementations need to be discussed [52]. In addition, adolescents are widely using recently introduced social media platforms that have not been considered yet for suicide preventive interventions (such as tik-tok) [53], and further studies are needed to fill this gap.

In general, the new and fast-developing technological tools (including language detection) might be part of suicide preventive strategies in adolescents in the future [52], supporting training for new strategies for suicide risk management [44]. However, beside technological developments, evidenced-based interventions to prevent suicidality in young people involve friends, families, school teachers, caregivers and clinicians [54]. It appears, therefore, to be more realistic that the new technologies will supplement existing strategies in the future rather than substituting for them. Nonetheless, such digital tools might be complementary with subjective approaches to suicide prevention, promoting a stronger connection with clinicians [55].

### Limitations

The findings of the present review should be interpreted in light of several limitations. These include, in particular, high levels of heterogeneity of methods and outcomes across the studies, incomplete reporting of suicidal ideation and attempts, sometimes-unclear diagnoses, the involvement of special populations (such as Australian indigenous people), and sampling from particular geographic areas or socioeconomic groups that may not generalize to others. Given the heterogeneity of methods and study outcomes, a quantitative synthesis of results was not feasible. Finally, most of the studies considered did not provide sufficient data to evaluate the efficacy of the intervention in reducing suicidal behavior among adolescents. Of note, a further limitation is that many new studies on technological tools are still not published, and so the present overview might have missed some of the existing digital tools that are being tested to prevent suicide in adolescents. Moreover, we focused our search on a few specific technological tools (telemedicine, mobile applications, and language detection), and we might have missed some of the new technological tools available.

## 5. Conclusions

The present findings suggest that new technologies provide well-tolerated and acceptable support for suicide prevention in adolescents. However, only limited data at the present time support the use of such interventions in clinical practice and prevention strategies. Overall, while there is some promise of these interventions for reducing suicidal ideation and attempts, whether this can be clinically useful remains unclear. Further studies are needed to test the efficacy of new technologies in suicide prevention for adolescents and young adults, and further studies are needed to compare validated face-to-face interventions with interventions based on new technologies.

## Figures and Tables

**Table 1:** Quality assessment of included studies.

Article	RCT	Method of Randomization	Treatment Allocation	Double Blinding	Power Calculation	Adherence	Continuous Exposure Variables	Times	Valid Outcome Measure	Drop-Out Rate
Aladag A.E. et al., 2018	N	Y	NA	NA	NR	NA	Y	N	N	N
Bailey E. et al., 2020	N	N	Y	N	Y	Y	Y	Y	Y	N
Brown R.C. et al., 2019	N	N	NA	N	NR	Y	N	N	Y	N
Chen R.Y. et al., 2017	N	N	Y	N	N	N	Y	N	Y	N
Dickter B. et al., 2019	N	N	Y	N	N	Y	Y	Y	Y	N
Downs J. et al., 2017	N	NA	NA	NA	N	NA	Y	N	Y	N
Franklin J. et al., 2016	Y	Y	Y	N	N	Y	Y	N	Y	N
Grant R.N. et al., 2018	N	N	N	N	NA	Y	Y	N	Y	NA
Grist R. et al., 2018	N	N	NA	N	NR	Y	Y	Y	Y	Y
Han J. et al., 2019	N	N	NA	N	N	Y	Y	N	Y	N
Hetrick S.E. et al., 2018	N	N	NA	N	N	Y	Y	Y	Y	N
Hill R.M. et al., 2016	Y	Y	Y	N	N	Y	Y	Y	Y	N
Kennard B.D. et al., 2018	N	Y	Y	N	Y	Y	Y	Y	Y	Y
McManama O’Brien K.H. et al., 2016	N	N	Y	N	N	N	N	N	N	N
Milton A.C. et al., 2019	N	Y	NA	N	N	N	N	N	Y	N
Ospina-Pinillos et al., 2018	N	Y	Y	NA	N	N	N	N	Y	NA
Owens C. et al., 2016	N	N	NA	NA	N	NA	N	N	N	NA
Robinson J. et al., 2016	N	N	N	N	N	Y	Y	Y	Y	Y
Runkle J.D. et al., 2020	NA	NA	NA	NA	N	NA	N	Y	N	N
Thabrew H. et al., 2019	Y	Y	Y	N	N	Y	Y	N	N	Y

Notes. N = No, NA = Not Applicable, NR = Not Reported, Y = Yes.

**Table 2:** Summary of reports include.

Article	Technology	Type of Article	Gender (Female %)	N. Participants	Target Group	Age Range	Diagnosis	Outcome	Intervention
Aladag A.E. et al., 2018	Language detection	Retrospective cohort study	/	785 (posts)	General	/	/	Suicidality	Prevention (self-guided)
Bailey E. et al., 2020	Telepsychiatry	Open-label single group trial	55%	20	General	16–25 (21.7 mean)	/	Feasibility, safety, acceptability and suicidal ideation	Prevention (self-guided/specialistic)
Brown R.C. et al., 2019	Language detection	Retrospective cohort study	87%	52	General	Mean age 16.6	/	Suicidal thoughts, acute suicidality	Prevention (self-guided)
Chen R.Y. et al., 2017	Telepsychiatry	Open-label single group trial	/	9	Clinical	adolescents	MDD,,break/>Autism Spectrum Disorders (ASD)	Response rate, suicidal behavior and ideation.	Prevention (self-guided)
Dickter B. et al., 2019	Telepsychiatry	Open-label single group trial	56.2	83	Clinical	14–21	MDD	Suicidal ideation	Prevention (self-guided)
Downs J. et al., 2017	Language detection	Retrospective cohort study	/	1906	Clinical	14–18	ASD	Suicidal ideation.	Postvention (self-guided)
Franklin J. et al., 2016	APP	RCT	80.7	114	General	mean age 23.02	/	Suicide plans and behavior.	Prevention (self-guided)
Grant R.N. et al., 2018	Language detection	Retrospective cohort study	/	63,252 (posts)	General	/	/	Latent topics related to suicide ideation.	Prevention (self-guided)
Grist R. et al., 2018	APP	Open-label single group trial	90	44	Clinical	12–17	MDD, Anxiety disorder	Suicidal behavior	Prevention (self-guided)
Han J. et al., 2019	Telepsychiatry	Open-label single group trial	92.5	43	General	16–25	/	Acceptability, suicidal ideation.	Postvention (specialist)
Hetrick S.E. et al., 2018	APP	Open-label single group trial	76.9	13	Clinical	18–25	MDD	Mood monitoring, suicidal ideation.	Prevention (self-guided)
Hill R.M. et al., 2016	Telepsychiatry	RCT	68.8	80	General	13–19	/	Perceived burdensomeness, thwarted belonginess, depressive symptoms	Prevention (specialist)
Kennard B.D. et al., 2018	APP	Randomized study	89.4	66	Clinical	12–18	MDD, Anxiety disorder	Suicidal ideation, behavior, treatment utilization and satisfaction	Postvention (self-guided)
McManama O’Brien K.H. et al., 2016	APP	Open-label single group trial	80.7	20	General	13–18	/	Acceptability, usability, suicidal ideation	Prevention (self-guided)
Milton A.C. et al., 2019	Telepsychiatry	Open-label single group trial	50	1400	General	16–25	/	Sexting, suicidal thoughts and behavior.	Prevention (self-guided)
Ospina-Pinillos et al., 2018	Telepsychiatry	Open-label single group trial	71.6	204	General	16–25	/	Online vs. face to face assessments	Postvention (specialist)
Owens C. et al., 2016	Telepsychiatry	Open-label single group trial	/	27	General	12–18	/	Self-harming behaviors	Prevention (self-guided, specialist)
Robinson J. et al., 2016	Telepsychiatry	Open-label single group trial	87.5	32	Clinical	14–18	MDD	Suicidal ideation, hopelessness and depression.	Prevention (self-guided, specialist)
Runkle J.D. et al., 2020	Telepsychiatry	Open-label single group trial	/	34.71	General	15–24	/	Help-seeking patterns	Prevention (self-guided)
Thabrew H. et al., 2019	Telepsychiatry	Randomized study	49	110	General	13–14	/	Completion times, detection rates, acceptability	Prevention (self-guided)
